# Differential Encoding of Odor and Place in the Mouse Piriform and Entorhinal Cortex

**DOI:** 10.1523/ENEURO.0026-25.2025

**Published:** 2025-10-07

**Authors:** Wilson Mena, Keeley Baker, Alon Rubin, Shaun Kohli, Yun Yoo, Brice Bathellier, Yaniv Ziv, Alexander Fleischmann, Shahab Rezaei-Mazinani

**Affiliations:** ^1^Université Paris Cité, Institut Pasteur, Unit of Neural Circuits Dynamics and Decision Making, Paris F-75015, France; ^2^Department of Neuroscience and Carney Institute for Brain Science, Brown University, Providence, Rhode Island 02912; ^3^Department of Brain Sciences, Weizmann Institute of Science, Rehovot 76100, Israel; ^4^Université Paris Cité, Institut Pasteur, AP-HP, INSERM, CNRS, Fondation Pour l'Audition, Institut de l’Audition, IHU reConnect, Paris F-75012, France; ^5^Center for Interdisciplinary Research in Biology (CIRB), Collège de France, Université PSL, CNRS, INSERM, Paris 75005, France; ^6^Département BEL, Mines Saint-Étienne, Centre CMP, Gardanne F-13541, France

**Keywords:** lateral entorhinal cortex, navigation, odor encoding, pirifrom cortex, place encoding, spatial information

## Abstract

The integration of olfactory and spatial information is critical for guiding animal behavior. The lateral entorhinal cortex (LEC) is reciprocally interconnected with cortical areas for olfaction and the hippocampus and thus ideally positioned to encode odor–place associations. Here, we used miniendoscopes to record neural activity in the mouse piriform cortex (PCx) and LEC. We show that in head-fixed mice, odor identity could be decoded from LEC ensembles but less accurately than from PCx. In male mice freely navigating a linear track, LEC ensemble activity at the odor ports was dominated by spatial information. Spatial position along the linear track could be decoded from LEC and PCx activity; however, PCx but not LEC exhibited strong behavior-driven modulation of positional information. Together, our data reveal that information about odor cues and spatial context is differentially encoded along the PCx–LEC axis.

## Significance Statement

For most animals, the sense of smell is essential for successfully navigating the environment to find food, shelter, and mates. However, how olfactory and spatial information is integrated in the brain to support olfactory-guided behaviors remains poorly understood. In mammals, candidate brain regions thought to support odor–place associations include the olfactory (piriform, PCx) cortex, entorhinal cortex, and hippocampus. We here characterize the activity of cells in the PCx and lateral entorhinal cortex (LEC) of freely moving mice in response to odor cues presented in a linear track. Using miniendoscope microscopy and population coding analyses, we find that information about odors, spatial location, and behavior is differentially encoded along the PCx–LEC axis.

## Introduction

Odor cues in the environment serve as important spatial landmarks for animal navigation. In mammals, information about odors is represented in the olfactory cortex, while the hippocampus plays a key role in the encoding of spatial information (O’Keefe and Dostrovsky, 1971; Wilson and McNaughton, 1993; Knierim et al., 2006; Stettler and Axel, 2009; Miura et al., 2012; Bolding and Franks, 2017; Eichenbaum, 2017; Roland et al., 2017). The olfactory cortex and hippocampus are strongly and reciprocally interconnected via the lateral entorhinal cortex (LEC), and the olfactory cortex, hippocampus, and entorhinal cortex form the mammalian allocortex, suggesting a conserved link between neural representations of odor and space throughout evolution (Shepherd, 2011; Knierim et al., 2014; Cleland and Linster, 2019).

Odors are detected by olfactory sensory neurons in the olfactory epithelium, and odor-evoked neural activity is initially transformed into spatiotemporal patterns of glomerular activity in the olfactory bulb (Mombaerts, 2004; Mori and Sakano, 2011). Olfactory bulb mitral and tufted cells transmit odor-evoked neuronal activity to multiple cortical areas for olfaction, including the piriform cortex (PCx) and LEC (Giessel and Datta, 2014; Cleland and Linster, 2019; Chen et al., 2021). The hippocampus has long been recognized for its role in encoding spatial and contextual information. Hippocampal place cells, for example, selectively fire when an animal traverses a particular location and are considered a neural substrate of spatial memory (O’Keefe and Dostrovsky, 1971; McNaughton et al., 2006; Eichenbaum, 2017; Takahashi, 2018).

Interestingly, recent findings have shown that odor cues at defined locations can serve as spatial landmarks and refine spatially dependent neuronal activity in the hippocampus (Radvansky and Dombeck, 2018; Fischler-Ruiz et al., 2021). On the other hand, in rats performing an olfactory navigation task, PCx neurons robustly encode spatial information about the environment (Poo et al., 2022). Together, these observations suggest that information about odors and their location in space is communicated across olfactory and hippocampal circuits.

The LEC is reciprocally interconnected with PCx and the hippocampus and a prime candidate for integrating olfactory and spatial information. LEC receives direct olfactory inputs via projections from olfactory bulb mitral cells and indirectly via PCx neurons (Giessel and Datta, 2014; Leitner et al., 2016; Bitzenhofer et al., 2022). The LEC receives spatial information via projections from hippocampus CA1 and subiculum and indirectly via extensive reciprocal connections within the entorhinal–hippocampal network (Kerr et al., 2007; Knierim et al., 2014; Igarashi, 2016). Therefore, a comparative analysis of odor-evoked and location-dependent neuronal activity along the PCx–LEC–hippocampal axis is critical for understanding neural circuit mechanisms for odor–place associations.

Here, we recorded neural activity in the PCx and LEC, in mice passively exposed to odors, and in mice exploring odor cues in a linear track. We found that odor identity information was more accurately encoded in PCx than in LEC, independent of behavioral state. Place information, in contrast, was accurately represented in both LEC and in PCx. However, the representations of symmetric positions along the track were highly correlated in PCx, suggesting more schema-like representations of distances and actions. Together, our data suggest that information about odor and place are differentially encoded along the PCx–LEC axis.

## Materials and Methods

### Mice

Adult C57BL/6J male mice (8–10 weeks old) were used in the study. All animals were grouped-housed with *ad libitum* access to food and water, in controlled temperature and humidity conditions, and exposed to conventional 12 h light/dark cycles. All procedures in the experimental protocol were approved by the French Ethical Committee (authorization APAFIS#2016012909576100). Experiments were conducted in accordance with EU Directive 2010/63/EU. All efforts were made to reduce animal suffering and minimize the number of animals needed to obtain reliable results.

### Virus injection

All surgeries were performed under ketamine (80 mg/kg)–xylazine (1 mg/kg) anesthesia. The animal's body temperature was maintained at 36°C using a feedback-based thermal blanket with a rectal probe (rodent Warmer X1, Stoelting). Mice were placed in a stereotaxic frame (David Kopf Instruments), and injections of 100 nl of GCaMP7s (pGP-AAV1-syn-jGCaMP7s-WPRE, Addgene plasmid #104487) diluted one-third in phosphate-buffered saline (PBS) were made at 50 nl/min at five locations separated by 200–250 µm. We used glass micropipettes and a manual injection system to deliver the virus; stereotaxic coordinates used for PCx were A/P, +0.5 mm; M/L, −3.5 mm, and D/V, −3.8 mm and for LEC were A/P, −3.15 mm; M/L, −4.15 mm, and D/V, −2.5 mm. After surgery, mice were singly housed for 1 or 2 weeks without any manipulation.

### GRIN lens implantation

Two to three weeks after the viral injection, a gradient refractive index (GRIN) lens of 0.5 mm diameter by 6.0 mm length (Inscopix) was implanted above the injection site to enable optical access to PCx or LEC neurons. First, a blunt needle was inserted and removed at 100–200 µm/min before the lens was implanted. The lens was sealed to the skull with dental cement (Super-Bond C&B, Sun Medical) and a low-viscosity composite (Flow-It ALC, Pentron). A head post, allowing transient head fixation of the animal to facilitate baseplate positioning and miniature microscope mounting, was attached to the skull with Pi-Ku-Plast HP36 and low-viscosity composite (Flow-It ALC, Pentron). After lens implantation, animals were singly housed to prevent damage to the implant. No animals displayed evident motor or behavior abnormalities after the surgery. Two to three weeks following the surgery, a baseplate for the nVista microscope (v2.0, Inscopix) was mounted onto the animal's head. In order to find a field of view (FOV) with responsive cells, an odor puff was presented before baseplate implantation. After 1 to 2 weeks, mice started pretraining sessions.

Animals were habituated to the nVista miniature microscope attached to their head before the experimental session (pretraining Phase 2; see below). GCaMP7s was excited with a light-emitting diode (LED) providing blue light at 475 mm, and fluorescence light was collected through a 535 ± 25 nm emission filter and a complementary metal-oxide semiconductor camera chip, embedded in the miniature microscope (Inscopix). Following 15–20 min of free exploration of the linear track, recordings of neuronal activity were acquired at 20 Hz with a 50 ms exposure time, for 30–90 min.

### Behavior

During the entire behavioral task period, food was available *ad libitum*, and animal weight was monitored daily. Water restriction (0.6–1 ml/d) was interleaved with a 12 h *ad libitum* supply overnight every Friday. Mice performed behavior 2–3 d per week.

Pretraining: the linear track consisted of a Plexiglass structure 100 cm long, 8 cm wide, and 9 cm high. After 2 consecutive days of water restriction, mice were trained for 7–11 d to run back and forth along the track by giving them ∼10 µl of water when nose poking on each end of the track. During pretraining, nose poking was followed immediately by odor presentation (1 s) and the water reward (1 s). Mice were kept under this regiment until they performed an average of 60 trials in 15 min on at least 2 consecutive days. After mice completed pretraining, a delay of 1 s between odor presentation and water reward was added, and a 3D-printed miniscope with similar dimensions and weight as the real one was implanted during training to habituate mice to run and nose poke with the miniscope attached.

### Calcium imaging and behavioral recordings

For experiments in the linear track, after pretraining, odorants (ethyl acetate, limonene, hexanal diluted to 0.3% v/v in mineral oil) were introduced. The FOV, focus, signal gain, LED intensity, and exposure time for GCaMP fluorescence were optimized for each mouse. FOVs selected during baseplate implantation were the same for freely moving recordings, and the same parameters for signal gain, LED intensity, and exposure time were used. After setting up optimal imaging parameters, mice were put back into their home cage 20–40 min for miniscope habituation before the beginning of the task. Miniscope recordings and nose-poking detections were synchronized using the Inscopix data acquisition box. A red LED light located next to the linear track was used to align the beginning and the end of calcium imaging and behavioral recordings.

For head-fixed recordings, mice were placed inside a modified Falcon tube and passively exposed to odor. Imaging parameters were adjusted to be the same between linear track and head-fixed regiments. The set of odorants, concentration, and duration of odor presentation was the same as for freely moving conditions. At the end of the recordings, the miniscope was removed, and mice were transferred to their home cage with *ad libitum* access to water and food. All brains were subsequently processed for histology.

### Histology

Histology was used to validate viral targeting of PCx and LEC. Mice were anesthetized with Euthasol (150 mg of pentobarbital/kg) and transcardially perfused with 10 ml of ice-cold PBS followed by 10 ml of 4% paraformaldehyde (PFA). Brains were dissected and postfixed overnight in 4% PFA at 4°C and transferred to PBS. Coronal sections (200 µm) were prepared using a vibrating-blade Leica VT100S Vibratome. Sections were rinsed in PBS and incubated in PBS/0.1% Triton X-100 and Neurotrace counterstain (1:1,000, Thermo Fisher Scientific) at 4°C overnight and then mounted on SuperFrost Premium microscope slides (Thermo Fisher Scientific, catalog #12-544-7) in Fluorescent Vectashield Mounting Medium (Vector). Confirmation of both lens position and GCaMP expression was obtained using a Nikon A1R-HD confocal microscope. Overall, the success rate for this surgery, including methods development, was ∼80%. Most common failures were (1) scratched/chopped GRIN lens due to the animal removing the lens protection cap and (2) excessive movement artifact or weak fluorescence signal on the FOV.

### Data structure

For all odor coding analyses, the output of Inscopix's CNMF-based cell segmentation software was a two-dimensional time series matrix, with cells in columns and calcium signals in rows. Head-fixed calcium signals consisted of six trials with 30 s duration, for four stimuli, pseudorandomly presented and with a 30 s intertrial interval. A 30 s trial contained 10 s prestimulus, 2 s odor response, and 18 s poststimulus phases. For freely moving calcium signals, a trial contained a 1 s prestimulus, 2 s odor response, and 1 s poststimulus phases. A total of 48 trials were organized into sets of 12 trials per odor, 6 trials at the right and 6 trials at the left odor port. For subsequent analyses, calcium traces were reorganized into a four-dimensional dataset (cells, time, trials, odors).

### Preprocessing

We smoothened calcium signals using a sliding window average of a length of five frames. Calcium signals had an arbitrary baseline. To normalize, we first calculated *F*_0_ by subtracting each signal from its mean value within an interval of 1 s before stimulus presentation. We then *z*-scored signals by dividing *F*_0_ calcium signals by the standard deviation of all trials and odors.

### Cellular analysis

We calculated the percentage of odor-responsive cells as the average of the fraction of active neurons across all trials for each odor. We selected activated neurons as those whose activity level was higher than the threshold of three times the standard deviation of the normalized baseline (1 s before the stimulation onset) and remained above this threshold for at least 500 ms. To quantify response magnitudes, we averaged the magnitude of the *z*-scored signals of the activated cells, over the 2 s odor-exposure period, across all trials of each odor, per mouse. Then, we calculated the average magnitude and standard deviation for all mice. Response duration was calculated as the time that a cell's activity exceeded the threshold of three times the standard deviation of the normalized baseline, for a minimum of 15 consecutive frames. We averaged response durations over all the cells, trials, and odorants.

### Population coding analysis

Pearson's correlation: we quantified the similarity of PCx and LEC odor responses using Pearson's correlation. We firstly calculated the average of calcium signals over the 2 s odor-exposure period. We then concatenated all the trials of each odor per all cells. We computed Pearson's correlation over all the cells across the ensemble of trials.

Odor classification: We quantified odor information encoded in PCx and LEC neural activity using a support vector machine (SVM) classifier with a linear kernel (fitcecoc function, MATLAB, MathWorks). The classifier predicted odor identity from neural response patterns in single trials. We used a leave-one-trial-out cross–validation approach, in which each trial was iteratively excluded from the training set and used as a test trial, while the remaining trials were used to train the classifier. Classification was performed over time using a 250 ms sliding window, moving frame by frame across the trial (sampling rate, 20 Hz), resulting in time-resolved decoding accuracy. For each time window, neural responses were averaged across frames to generate features, and decoding was performed independently for each window. Predicted class labels were compared with the true odor labels to compute the probability of correct classification at each time point. Decoding accuracy was averaged across trials (*N* = 6 in head-fixed and *N* = 12 in freely moving per odor, three odors) to produce a time-resolved decoding curve, and overall accuracy was calculated by averaging across the 2 s odor-exposure period. The chance level for correct classification was 33.3%. The analysis was implemented in an object-oriented MATLAB framework, with a master function coordinating trial iteration, sliding window computation, SVM model training, prediction, and accuracy calculation.

Ensemble size classification: To determine classification accuracy as a function of the neural ensemble size, we trained the SVM classifier (as described above) on odor responses from randomly selected subsets of neurons. Starting with a group of five randomly selected cells, we increased the ensemble size in steps of five cells by adding additional randomly selected neurons, repeating this until all recorded cells were included. For each ensemble size, odor classification was performed using leave-one-trial-out cross–validation, with decoding accuracy computed over time using a 250 ms sliding window at a sampling rate of 20 Hz. This process was iterated 100 times, each iteration selecting a new random set of neurons at each ensemble size. For each ensemble and iteration, we extracted the maximum decoding accuracy within the 2 s odor stimulus interval from the time-resolved decoding curve. To visualize classification performance, we computed a histogram of maximum decoding accuracy values across iterations for each ensemble size and normalized the histogram by the number of iterations to obtain the probability distribution of maximum decoding accuracy per ensemble size. These distributions were displayed as a heatmap, with classification accuracy shown as a function of the ensemble size.

### Statistical analysis

All quantification and statistical analysis were performed using MATLAB R2021 a. Statistical assessment was performed using nonparametric tests, as reported in the text. In all figures, error bars display standard deviation. The 95% confidence interval was used in Extended Data Figure 2-1 for the error's shaded area.

Binomial test: To evaluate whether odor decoding accuracy at each time point was significantly greater than chance, we used a binomial test. At each time point, the number of correct classifications, obtained using a SVM decoder applied to LEC and PCx activity within a 2 s time interval, was tested against the total number of decoded trials.

The probability of obtaining exactly *k* successes (correct classifications) in *n* trials with a chance-level probability *p* is given by the binomial distribution as follows:

$$P(k)=f(k,n,p)=\left(\begin{array}{c} n\\ k \end{array}\right)p^{k}(1-p{)}^{n-k},$$

where *k* is the number of correct classifications (successes), *n* is the number of trials (here, 36), and *p* is the expected chance-level probability of success (here, *p* = 1/3, corresponding to three uniformly distributed stimuli presented in the task).

The *p* value was computed as the probability of observing *k* or more successes under the binomial distribution. This yielded a time series of *p* values, indicating at each time point whether decoding accuracy was significantly greater than chance.

### Encoding of behaviorally relevant spatial information

For the linear track, we constructed a maximum correlation decoder to infer the position of the mouse at each frame based on the relations between the neuronal activity patterns during the exploration of the linear track; either in the same or the opposite running direction. For cross-validation, frames on odd trials were decoded solely based on data from even trials and vice versa. We divided the 100 cm linear track into 24 bins, and we defined the activity pattern of each spatial bin as the vector of the activity rate per neuron at that bin. For each frame, we inferred the mouse's position by maximizing the correlation of the activity pattern in that frame with the activity pattern of a bin (namely, the decoded position was the bin whose activity pattern had the highest correlation with that of the decoded frame). For this analysis, we considered only running epochs (velocity > 2 cm/s and excluding the last two bins at each end of the tracks). For the evaluation of the decoding accuracy, for each frame, we calculated the squared difference between the actual and the decoded positions and took the mean over all the frames considering only running epochs.

### Code accessibility

The MATLAB code described in the paper is freely available online at https://gitlab.com/fleischmann-lab/papers/odor-coding-analysis. The code is available as Extended Data.

10.1523/ENEURO.0026-25.2025.d1 Data 1 **Odor encoding analysis codes** This MATLAB codebase provides a pipeline for processing and analyzing calcium imaging data from freely-moving and head-fixed odor-detection experiments. The analysis includes event-aligned calcium trace extraction, normalization, dimensionality reduction, classification (KNN and SVM), as well as single-cell and population response profiling. Additional tools support alignment across behavioral contexts to test context-dependent odor coding. The modular design enables flexible and reproducible analysis across multiple datasets. Download Data 1, ZIP file.

## Results

### Odor representations in LEC and PCx in head-fixed mice

**Figure 1.**
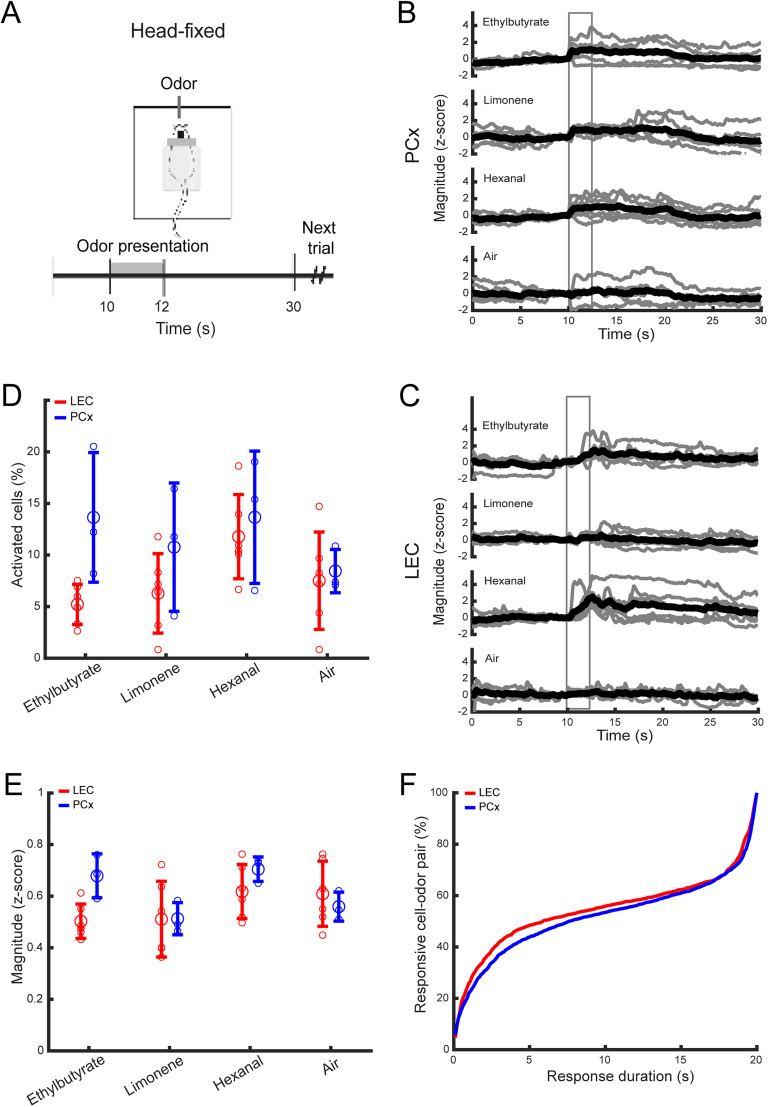
Calcium imaging of odor-evoked activity in LEC and PCx of head-fixed mice. ***A***, Head-fixed experimentation scheme and trial structure. ***B***, ***C***, Example traces of six trials and their average, from one PCx and one LEC neuron, in response to three monomolecular odorants and clean air. Gray box, odor-exposure window. ***D***, The percentage of neurons activated in LEC (red) and PCx (blue; Mann–Whitney *U* test, averaged per mice over all odors; *p* = 0.03). Large circles, Average across six LEC mice and three PCx mice per stimulus; small circles, data points from individual mice and stimuli; error bar, standard deviation. ***E***, Response magnitude in LEC and PCx, averaged across six (LEC) and three (PCx) mice (Mann–Whitney *U* test, *p* = 0.19). Large circles, average, small circles, data points from individual mice; error bar, standard deviation. ***F***, The percentage of responsive cell–odor pair as a function of response duration (in second) in LEC and PCx (Kolmogorov–Smirnov test, *p* = 0.25).

We stereotaxically injected adeno-associated virus expressing the calcium indicator GCaMP7 (Dana et al., 2019) into the LEC and PCx of adult (6–8-week-old) mice. A GRIN lens was implanted above the injection site, and all behavioral and imaging experiments were performed between 6 and 12 weeks after lens implantation (Extended Data Fig. 1-1). Visual inspection of individual imaging sites revealed well-identifiable cells exhibiting robust fluctuations in fluorescence (Fig. 1*A*–*C*). Cell segmentation was performed using the Inscopix CNMFe algorithm.

10.1523/ENEURO.0026-25.2025.f1-1 Figure 1-1 **GCaMP7 injection and GRIN lens implantation sites in LEC and PCx** Top left: Atlas schematics of coordinates of viral injection and GRIN lens implantation for the piriform cortex. Top middle: Representative GCaMP7s injection and GRIN lens implantation sites in the piriform cortex. Top right: An enlarged view of the GCaMP7s injection and GRIN lens sites in the piriform cortex. Scale bar: 200 µm represents the approximate imaging distance below the GRIN lens. Bottom left: Atlas schematics of coordinates of viral injection and GRIN lens implantation of the LEC. Bottom middle: Representative GCaMP7s injection and GRIN lens implantation sites in the LEC. Bottom right: An enlarged view of the GCaMP7s injection and GRIN lens sites in LEC. Scale bar: 200 µm represents the approximate imaging distance below the GRIN lens. Download Figure 1-1, TIF file.

To compare basic odor response properties in LEC and PCx, we initially exposed head-fixed mice to ethylbutyrate, limonene, hexanal (0.3% v/v in mineral oil), and clean air as a control (six trials each, 30 s intertrial interval; see Materials and Methods; Fig. 1*A*–*C*). We analyzed a total of 1,235 LEC cells in six mice (mean ± SD, 205.8 ± 76.4) and a total of 611 PCx cells in three mice (mean ± SD, 203.6 ± 74.5).

We found that the percentage of odor-responsive cells in LEC was significantly lower than in PCx (LEC, mean ± SD, 7.69% ± 2.88; PCx, mean ± SD, 11.62% ± 2.52; Mann–Whitney *U* test; *p* = 0.03; Fig. 1*D*). Furthermore, the response magnitude of cells in LEC appeared slightly lower than in PCx [LEC, mean ± SD, 0.55 ± 0.05 (*z*-score), PCx, mean ± SD, 0.61 ± 0.09 (*z*-score); *p* = 0.19; Fig. 1*E*], and the distribution of odor response duration was slightly shifted toward shorter responses in LEC than in PCx (Kolmogorov–Smirnov test; *p* = 0.25; Fig. 1*F*). These data suggest that odor responses in LEC are less robust than in PCx.

**Figure 2.**
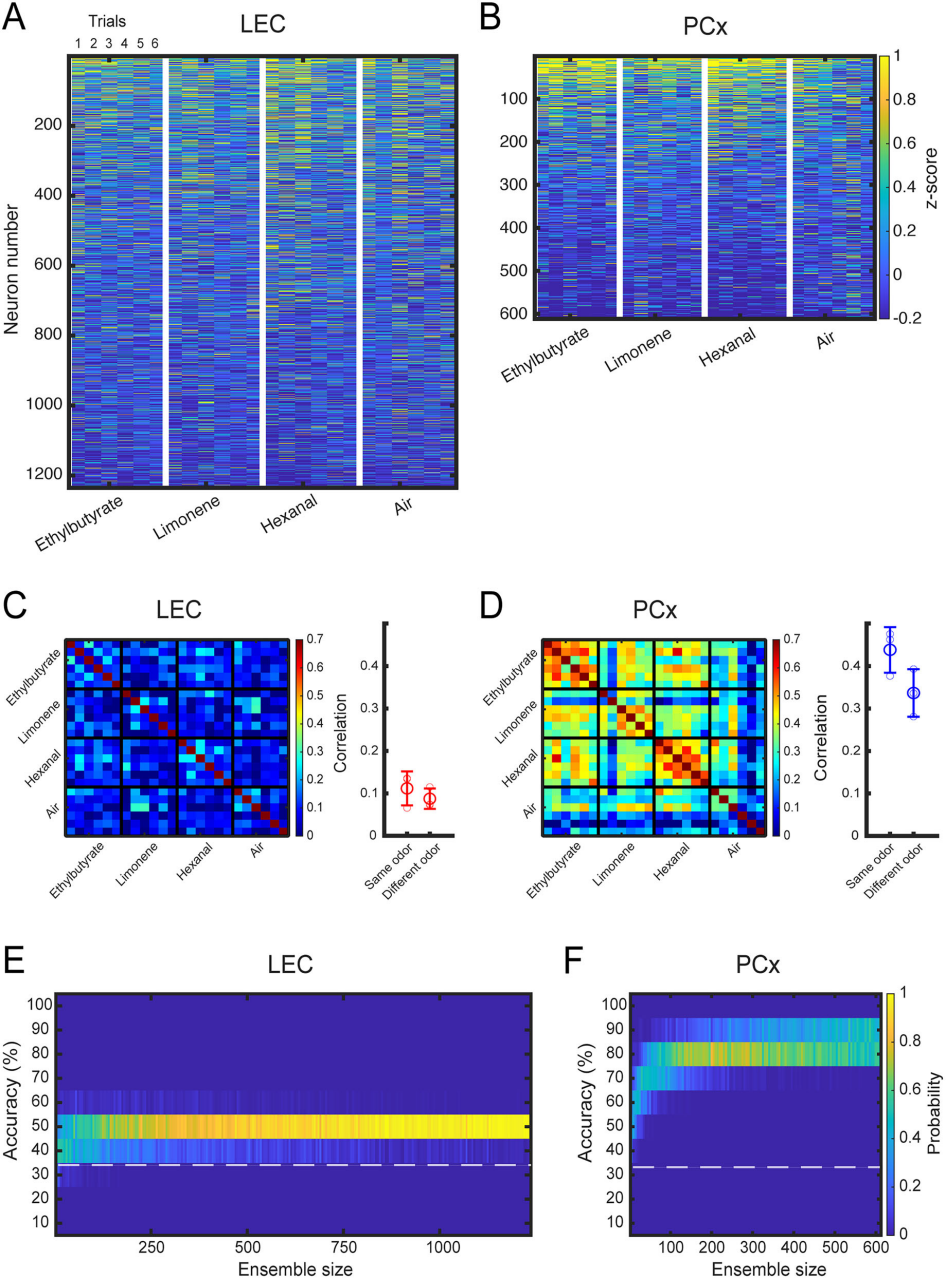
Representations of odor identity in LEC and PCx of head-fixed mice. ***A***, Heatmap of neural activity in LEC. Each stimulus rectangle (separated by a white vertical line) is subdivided into six columns of smaller squares, each representing a single trial per cell. ***B***, Heatmap of neural activity in PCx. Each stimulus rectangle (separated by a white vertical line) is subdivided into six columns of smaller squares, each representing a single trial per cell. ***C***, Left, Similarity matrix representing the pairwise correlation coefficients between neuronal activity population response vectors in LEC. Data obtained from six pooled mice (2 s odor-exposure window). Every small square represents a trial. A group of six trials constitutes an odor. Right, Correlation of odor responses between repeat exposure to the same versus different odorants (difference between same and different odor correlation; Mann–Whitney *U* test; *p* = 0.18). Large circles, average; small circles, data points from individual mice; error bar, standard deviation. ***D***, Left, Similarity matrix for neuronal activity population response vectors in PCx. Data obtained from three pooled mice (2 s odor-exposure window). Every small square represents a trial. A group of six trials constitutes an odor. Right, correlation of odor responses for same versus different odorants (difference between same and different odor correlation; Mann–Whitney *U* test; *p* = 5 × 10^−6^). Large circles, average; small circles, data points from individual mice; error bar, standard deviation. ***E***, Accuracy of odor identity classification in pseudopopulations of the increasing size in LEC. Distribution of the accuracy of odor classification using a linear SVM classifier trained on randomly sampled LEC ensembles of the increasing size (2 s odor-exposure window). Total number of neurons, *n* = 1,235. The white-dotted line shows the chance level. ***F***, Accuracy of odor identity classification in pseudopopulations of the increasing size in PCx. Distribution of the accuracy of odor classification for PCx ensembles of the increasing size (2 s odor-exposure window). Total number of neurons, *n* = 611. The white-dotted line shows the chance level.

We next asked whether LEC and PCx odor representations differ in how they encode odor identity information. We first compared the similarities of odor-evoked activity across trials and odorants. We calculated the pairwise cross-correlations between single-trial population response vectors during the 2 s of odor exposure, pooled across LEC and PCx imaging sites. Correlation matrices indicated that in LEC, overall trial correlations were lower than in PCx (Fig. 2*A*–*D*; Extended Data Fig. 2-1). Moreover, in contrast to PCx, repeated exposure to the same odorants did not elicit more correlated population activity than exposure to different odorants (LEC same odor correlation, mean ± SD, 0.11 ± 0.04; different odor correlation, mean ± SD, 0.09 ± 0.02; *p* = 0.18; PCx same odor correlation, mean ± SD, 0.44 ± 0.05; different odor correlation, mean ± SD, 0.34 ± 0.06; *p* = 5 × 10^−6^; Fig. 2*C*,*D*).

10.1523/ENEURO.0026-25.2025.f2-1 Figure 2-1 **Dynamics of odor identity encoding in LEC and PCx in head fixed (A, B)** **Top panel:** Accuracy of odor identity classification in LEC (A) and PCx (B) over time, for head-fixed mice. Time 0 indicates odor valve opening (vertical gray dashed line). Odor encoding is less accurate and delayed in LEC compared to PCx. The horizontal gray dashed line represents the chance level (1/3). Shaded area indicates 95% confidence intervals for the mean. **Middle panel:** Time-resolved binomial test of classification accuracy. The plots show the evolution of p-values over time (computed in time frames of 250 ms), testing whether decoding performance exceeds the chance level of 1/3. The vertical gray dashed line indicates odor valve opening (time = 0), and the horizontal gray dashed line marks the significance threshold (*p* = 0.05). Curve segments falling below this threshold indicate periods of statistically significant decoding. In (A), the LEC curve shows a ∼1-second delay before decoding becomes significant, whereas in (B), the PCx curve shows that odor encoding becomes significant immediately after odor presentation, with no apparent delay. **Bottom panel:** Confusion matrix summarizing the performance of the SVM classifier trained to discriminate the odorants. Decoding accuracy for each odor, averaged over a 2-second time window. LEC’s confusion matrix shows that classifier accuracy for ethylbutyrate reaches 69%, while limonene is classified at 54%. This accuracy for PCx is above 81% for all 3 odors. **(C)** Left: Similarity matrix representing the pairwise correlation coefficients between neuronal activity population response vectors in LEC. Data obtained from 6 pooled mice (5 sec odor-exposure window). Every small square represents a trial. A group of 6 trials constitutes an odor. Right: correlation of odor responses between repeat exposure to the same versus different odorants. Large circles: average, small circles: data points from individual mice; bar: standard deviation. **(D)** Left: Similarity matrix for neuronal activity population response vectors in PCx. Data obtained from 3 pooled mice (5 sec odor-exposure window). Every small square represents a trial. A group of 6 trials constitutes an odor. Right: correlation of odor responses for same versus different odorants. Large circles: average, small circles: data points from individual mice; bar: standard deviation. The extension of the odor exposure window to 5 seconds resulted in the decrease of correlation level. **(E)** Accuracy of odor identity classification in pseudo-populations of increasing size in LEC. Distribution of the accuracy of odor classification using a linear SVM classifier trained on randomly sampled LEC ensembles of increasing size (5 sec odor-exposure window). Total number of neurons: n = 1235. The white dotted-line shows the chance level. **(F)** Accuracy of odor identity classification in pseudo-populations of increasing size in PCx. Distribution of the accuracy of odor classification for PCx ensembles of increasing size (5 sec odor-exposure window). Total number of neurons: n = 611. The white dotted-line shows the chance level. The extension of the odor exposure window to 5 seconds led to a lower accuracy probability in ensemble-size classifications in LEC and PCx. Download Figure 2-1, TIF file.

To compare the accuracy of odor identity coding in LEC and PCx, we next used a linear SVM classifier (see Materials and Methods). Building classifiers with increasing numbers of randomly selected cells, we found that for similar ensemble size, average classification accuracy was significantly lower in LEC than in PCx (the matched pseudopopulation size of 610 neurons; LEC, mean ± SD, 43.5% ± 2.5; PCx, mean ± SD, 80% ± 2.9; *p* = 2.5 × 10^−34^; Fig. 2*E*,*F*; Extended Data Fig. 2-1*A*,*B*). Furthermore, while odor decoding accuracy in PCx reached significant performance shortly after odor onset (Extended Data Fig. 2-1*B*), classification accuracy in LEC reached significance only ∼1 s after odor exposure (Extended Data Fig. 2-1*A*).

### Odor representations in LEC and PCx in freely moving mice

**Figure 3.**
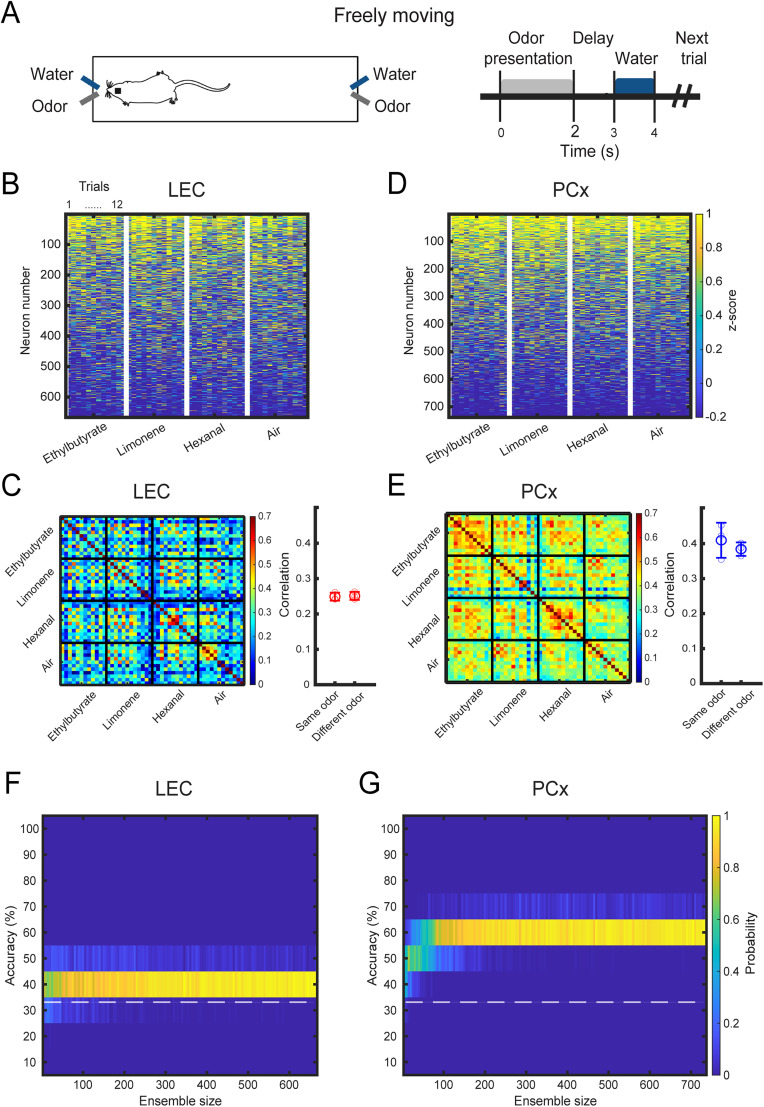
Representations of odor identity in LEC and PCx of freely moving mice. ***A***, Freely moving experimentation scheme and trial structure. ***B***, Heatmap of neural activity in LEC. Each stimulus rectangle (separated by a white vertical line) is subdivided into 12 columns of smaller squares, each representing a single trial per cell. ***C***, Left, Similarity matrix representing the pairwise correlation coefficients between neuronal activity population response vectors in LEC (2 s odor-exposure window). Data obtained from three pooled mice. Every small square represents a trial. A group of 12 trials constitutes an odor. Right, Correlation of odor responses between repeat exposure to the same versus different odorants (LEC; difference between same and different odor correlation; Mann–Whitney *U* test; *p* = 0.64). Large circle, average; small circles, data points from individual mice; error bar, standard deviation. ***D***, Heatmap of neural activity in PCx. Each stimulus rectangle (separated by a white vertical line) is subdivided into 12 columns of smaller squares, each representing a single trial per cell. ***E***, Left, Similarity matrix for neuronal activity population response vectors in PCx (2 s odor-exposure window). Data obtained from four pooled mice. Every small square represents a trial. A group of 12 trials constitutes an odor. Right, Correlation of odor responses for same versus different odorants (difference between same and different odor correlation; Mann–Whitney *U* test; *p* = 3.2 × 10^−3^). Large circle, Average; small circles, data points from individual mice; error bar, standard deviation. ***F***, Accuracy of odor identity classification in pseudopopulations of the increasing size in LEC. Distribution of the accuracy of odor classification using a linear SVM classifier trained on randomly sampled LEC ensembles of the increasing size (2 s odor-exposure window). Total number of neurons, *n* = 666. The white-dotted line shows the chance level. ***G***, Accuracy of odor identity classification in pseudopopulations of increasing size in PCx. Distribution of the accuracy of odor classification for PCx ensembles of the increasing size (2 s odor-exposure window). Total number of neurons, *n* = 738. The white-dotted line shows the chance level.

We next asked how odor information was represented in LEC and PCx when freely moving mice sampled odorants at odor ports in a linear track (Movie 1). Mice were trained to sample an odorant at an odor port at the end of the track, where odor exposure was triggered when mice poked the odor port. Odor exposure lasted for 2 s, followed by a 1 s delay before the water reward was delivered. Mice then moved to the other odor port to initiate the next trial (Fig. 3*A*). Behavior and neuronal activity were recorded for a total of 48 trials: 12 trials of each odorant (ethylbutyrate, limonene, hexanal, clean air) 6 trials at each odor port (see Materials and Methods).

**Movie 1.** Freely moving experiment along a linear track. A freely moving mouse performing an odor detection task in a linear track. [View online]

10.1523/ENEURO.0026-25.2025.f3-1 Figure 3-1 **Dynamics of odor identity encoding in LEC and PCx in freely moving (A, B) Top panel:** Accuracy of odor identity classification in LEC (A) and PCx (B) over time, for freely-moving mice. Time 0 indicates odor valve opening (vertical gray dashed line). Odor encoding is less accurate and delayed in LEC compared to PCx. The horizontal gray dashed line represents the chance level (1/3). Shaded area indicates 95% confidence intervals for the mean. **Middle panel:** Time-resolved binomial test of classification accuracy. The plot shows the evolution of the p-value over time (computed in time frames of 250 ms), testing whether decoding performance exceeds the chance level of 1/3. The vertical gray dashed line indicates odor valve opening (time = 0), and the horizontal gray dashed line marks the significance threshold (*p* = 0.05). Curve segments falling below this threshold indicate periods of statistically significant decoding. In (A), the LEC curve shows a ∼2-second delay before decoding becomes significant, whereas in (B), the PCx curve shows a shorter delay of 0.65 seconds before odor encoding becomes significant. **Bottom panel:** Confusion matrix summarizing the performance of the SVM classifier trained to discriminate the odorants. Decoding accuracy for each odor, averaged over a 2 second time window. The confusion matrix shows that classifier accuracy for ethylbutyrate reaches 55%, while limonene is classified at 45%, further supporting that odor-specific signals are present in LEC. This accuracy for PCx is above 90% in case of ethylbutyrate and hexanal, and 70% for limonene. Download Figure 3-1, TIF file.

We analyzed a total of 666 LEC cells in three mice (mean ± SD, 222 ± 91) and a total of 738 PCx cells in four mice (mean ± SD, 184 ± 118). Correlation matrices indicated that overall cross-trial correlations during the 2 s odor presentation in LEC increased in freely moving mice compared with head-fixed mice (Fig. 3*B*,*C*). Furthermore, neural activity patterns in response to the same odorant were not more similar to each other than neural activity patterns in response to different odorants (same odor correlation, 0.24 ± 0.01; different odor correlation, 0.25 ± 0.01; *p* = 0.64; Fig. 3*C*). PCx activity patterns similarly exhibited an overall increase in cross-trial correlations, independent from the odor stimulus (same odor correlation, 0.41 ± 0.05; different odor correlation, 0.38 ± 0.02; *p* = 3.2 × 10^−3^; Fig. 3*D*,*E*). However, a linear SVM classifier accurately predicted odor identity in PCx, while classifier performance in LEC was much inferior and remained close to the chance level for the 2 s window after odor presentation (the matched pseudopopulation size of 665 neurons; LEC, mean ± SD, 36.8% ± 1.8; PCx, mean ± SD, 57.1% ± 1.8; *p* = 3.3 × 10^−19^; Fig. 3*F*,*G*). Furthermore, while LEC decoding significantly exceeded the chance-level performance only ∼2 s after odor onset (Extended Data Fig. 3-1*A*), classification accuracy in PCx reached significance much earlier, <1 s after onset (Extended Data Fig. 3-1*B*). Together, these data suggest that odor identity coding in LEC is less accurate and delayed relative to neuronal coding in PCx, independent of whether mice are passively exposed to odors or actively sampling odors at odor ports in a linear track.

### Distinct representations of spatial location in LEC and PCx

The LEC receives strong inputs from the hippocampus and has been implicated in the integration of spatial and nonspatial information (Igarashi et al., 2014; Knierim et al., 2014; Vandrey et al., 2020). Furthermore, recent data show that in addition to odor information, spatial information is also encoded in the posterior PCx of rats (Poo et al., 2022). We therefore next examined the representation of spatial information in LEC and PCx.

**Figure 4.**
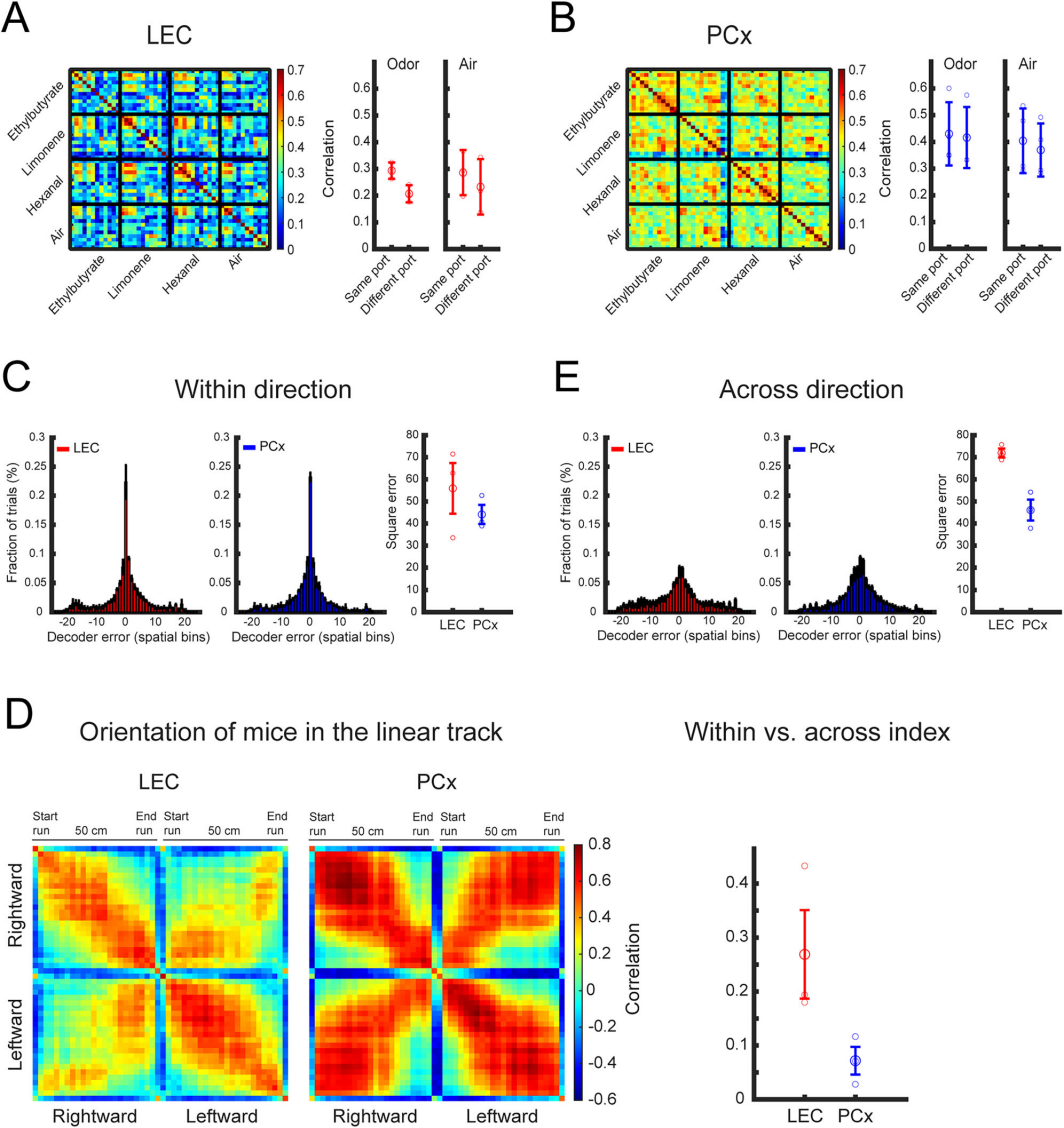
Representations of spatial location in LEC and PCx of freely moving mice. ***A***, Left, Similarity matrix representing the pairwise correlation coefficients between neuronal activity population response vectors in LEC (2 s odor-exposure window), reorganized in trials at the left odor port (first 6 trials) and trials at the right odor port (second 6 trials). Right, Correlation of neuronal activity at the left odor port versus the right odor port, averaged across different odorants (difference between same and different odor port; Mann–Whitney *U* test; *p* = 1.2 × 10^−7^). Here, each odor square is subdivided into 12 smaller squares, each representing a single trial. These are split into two groups of six trials corresponding to the right and left sides of the linear track. The air shows the control (difference between same and different air port; Mann–Whitney *U* test; *p* = 0.02). Large circle, average; small circles, data points from individual mice; error bar, standard deviation. ***B***, Left, Similarity matrix for neuronal activity population response vectors in PCx (2 s odor-exposure window), reorganized in trials at the left and right odor port. Right, Correlation of neuronal activity at the left versus right odor port, averaged across different odorants (difference between same and different odor port; Mann–Whitney *U* test; *p* = 0.22). Here, each odor square is subdivided into 12 smaller squares, each representing a single trial. These are split into two groups of six trials corresponding to the right and left sides of the linear track. The air shows the control (difference between same and different air port; Mann–Whitney *U* test; *p* = 0.12). Large circle, average; small circles, data points from individual mice; error bar, standard deviation. ***C***, Distributions of the decoder's errors in inferring the position of mice along the linear track. The decoder was trained and tested on data from running in the same direction. ***D***, Pearson's correlation between ensemble activity patterns across all spatial locations on the linear track on both directions, for data recorded in the LEC (left) and PCx (right). Ensemble activity patterns were calculated based on concatenated epochs from a given location, separated according to the two running directions. ***E***, Distributions of the decoder's errors in inferring the position of mice along the linear track. The decoder was trained on data from running in one direction and tested on data from running in the other direction (trajectory decoding difference; one-tailed *t* test; *p* = 0.04).

We reorganized cross-trial correlations in blocks of trials at each odor port, and we observed that in LEC, population activity was more highly correlated for trials at the same odor ports, independent of odor identity (same port correlation, 0.29 ± 0.03; different port correlation, 0.2 ± 0.03; *p* = 1.2 × 10^−7^). This odor port-specific correlation structure was not observed in PCx (same port correlation, 0.43 ± 0.1; different port correlation, 0.41 ± 0.1; *p* = 0.22; Fig. 4*A*,*B*). Similar results were obtained for trials with clean air (LEC, same port, 0.29 ± 0.08; different port, 0.23 ± 0.1; *p* = 0.02; PCx, same port, 0.4 ± 0.12; different port, 0.37 ± 0.1; *p* = 0.12).

We next tested the accuracy with which spatial position across the length of the linear track was represented in LEC and PCx. We defined a population vector for each position as the mean activity of each neuron at that position, and for each time frame, we identified the position for which the population vector had the highest Pearson's correlation (see Materials and Methods). We found that decoders at single frame resolution demonstrate similarly high performance for both brain regions (Fig. 4*C*). However, the analysis of the correlation structure of neuronal representations suggested differences in the encoding of spatial information relative to running direction. In LEC, the representation of a given position along the linear track was distinct for each running direction. In contrast, in PCx, the representation of opposite (symmetric) positions along the linear track was highly similar across running direction, suggesting a representation of the trajectory phase relative to the odor ports (Fig. 4*D*). Consistent with this observation, we found that decoding of the trajectory phase was significantly more accurate in PCx than in LEC [differentiation index = (corr. same − corr. different)/(corr. same + corr. different); LEC, 0.27 ± 0.14; PCx, 0.07 ± 0.04; one-tailed *t* test; *p* = 0.04; Fig. 4*E*].

Together, these results reveal that spatial position along the linear track is accurately represented in LEC and PCx activity. However, LEC represents position (trajectory phase) in conjunction with running direction, while PCx encodes position is a more schema-like manner, independent of running direction.

## Discussion

Memories of odors and places can be tightly intertwined, and odor–place associations are essential for animal navigation. The olfactory (piriform, PCx) cortex, LEC, and hippocampus form an evolutionarily conserved neural circuit that is ideally positioned to support odor–place associations (Haberly, 2001; Eichenbaum, 2017). We here recorded odor-evoked neural activity in PCx and LEC, using miniendoscope calcium imaging. We compared odor representations in head-fixed mice passively exposed to odor and in mice freely sampling odors at odor ports in a linear track. We found that odor identity was more accurately encoded in PCx than in LEC. Spatial information was accurately encoded in both LEC and in PCx. LEC encoded spatial position regardless of its immediate behavioral implications, which are differentially representing the same odor at different edges and differentially representing the same position along the linear track for different running directions (similarly to neuronal representation in classical spatial brain regions; Markus et al., 1995; Brun et al., 2008; Sheintuch et al., 2020). In contrast, PCx represented odor indifferently of animal's position and preferentially represented trajectory phase, regardless of running direction, which is positional information that is more directly relevant to behavior. Together, our data suggest that olfactory and spatial information is differentially processed within cortical olfactory circuits.

### Neural representations of odor identity in LEC are less accurate and delayed compared with PCx

The fraction of odor-responsive neurons and their response magnitudes were higher in PCx than in LEC, and responses were more prolonged. PCx neural activity patterns in response to re-exposure to the same odorant were more similar to each other than the neural activity patterns evoked by different odorants. In contrast, in LEC, odor-evoked neural activity was not correlated with odor identity. Finally, the accuracy of a linear SVM classifier in predicting odor identity from odor-evoked neural activity patterns was substantially higher in PCx than in LEC and emerged much earlier after odor presentation. Interestingly, the delay between the emergence of odor identity information in the two brain regions was similar across behavioral conditions (head-fixed and freely behaving mice), consistent with the possibility that LEC inherits odor-related information from PCx. Prior work has reported more accurate odor encoding in LEC than observed here (Woods et al., 2020; Bitzenhofer et al., 2022; Jun et al., 2024). The observed differences likely reflect differences in recording technology including temporal resolution, task design, and behavior. We here directly compared neural activity in PCx and LEC under the same experimental conditions. Thus, the relative differences in odor identity coding we observe likely reflect inherent differences between PCx and LEC.

Taken together, our results show that odor identity encoding was more robust in PCx than in LEC.

This observation is consistent with well-established differences in neural circuit connectivity: PCx is the main target of olfactory bulb mitral and tufted cells. In contrast, LEC receives fewer direct olfactory bulb inputs, only from mitral cells (Xu and Wilson, 2012; Giessel and Datta, 2014; Cleland and Linster, 2019). Our data thus suggest that the robust reciprocal connections between PCx and LEC are likely to convey higher-order odor features rather than information about odor identity alone.

### Task engagement does not improve odor coding in LEC

LEC is thought to integrate multimodal sensory and behavioral signals (Kerr et al., 2007; Knierim et al., 2014; Eichenbaum, 2017; Sugar and Moser, 2019), suggesting that LEC odor representations may be modulated by sensory and behavioral context. We therefore asked whether accurate odor identity representations in LEC require the active engagement of mice in a behavioral task.

We recorded odor-evoked activity while freely moving mice sampling odorants at odor ports in a linear track. We observed that odor-evoked neural activity at odor ports in the linear track was more highly correlated than under head-fixed conditions. However, correlated neural activity did not represent odor-specific information and was thus likely driven by behavioral and contextual signals at the odor ports. We found that the accuracy of odor identity coding in LEC was not increased with task engagement. Instead, the emergence of an accurate odor identity code was delayed, both in LEC and PCx, relative to head-fixed condition. Thus, sampling odors at odor ports in a linear track did not improve overall odor coding in LEC, relative to PCx. In our task, however, odor cues did not carry task-relevant information. In future experiments, it will be interesting to test whether tasks that rely on the specific association of odor with contextual spatial information engage LEC to represent odor information more accurately.

### Encoding of behaviorally relevant spatial information

Within the spatial and temporal limits of our experimental design, we found that the mouse's position along the linear track could be decoded from LEC and PCx activity with similar accuracy. Our results extend the recent discovery of spatially tuned cells in the posterior PCx of rats (Poo et al., 2022). Interestingly, however, our data suggest that LEC and PCx differ in how they represent behaviorally relevant spatial information.

We found that in LEC, the representation of a given position was distinct for each running direction, i.e., the representation of the same position differed for rightward and leftward running trajectories. In contrast, in PCx, the representation of symmetric positions along the linear track for opposite running directions was highly correlated, suggesting that PCx activity represented the position of the mouse relative to the start and end points of each track traversal, regardless of the running direction (i.e., the trajectory phase). This representation may reflect the reward expectation or the motor plan of stereotypic running a fixed distance. This transformation of positional information into behaviorally relevant positional information is reminiscent of our recent findings in the hippocampus and anterior cingulate cortex (Rubin et al., 2019). Determining the odor, spatial and behavioral tuning properties of individual PCx, LEC, and hippocampal neurons will be critical for understanding the neural computations underlying odor–place associations. More generally, the ability to record from large ensembles of the PCx, LEC, and hippocampus neurons in freely moving mice provides a powerful approach for elucidating neural circuit mechanisms for the integration of sensory and contextual information.

## Data Availability

We developed a MATLAB codebase for data analysis. The codebase is publicly available online at https://gitlab.com/fleischmann-lab/papers/odor-coding-analysis.

## Synthesis

Reviewing Editor: James Howard, Brandeis University Decisions are customarily a result of the Reviewing Editor and the peer reviewers coming together and discussing their recommendations until a consensus is reached. When revisions are invited, a fact-based synthesis statement explaining their decision and outlining what is needed to prepare a revision will be listed below. The following reviewer(s) agreed to reveal their identity: Izumi Fukunaga, Anne-Marie Oswald. Note: If this manuscript was transferred from JNeurosci and a decision was made to accept the manuscript without peer review, a brief statement to this effect will instead be what is listed below.

The authors have adequately addressed the concerns raised by reviewers. This is particularly true with respect to clarifying analysis methods and presentation. Concerns about interpretation of findings have also been clarified in both figures and the discussion. This work makes a valuable contribution to the field.
