# Supplementary figures and images for: Differential Encoding of Odor and Place in the Mouse Piriform and Entorhinal Cortex
Source: eNeuro. 2025 Oct 7;12(10):ENEURO.0026-25.2025. doi: 10.1523/ENEURO.0026-25.2025 (PMC12507510; doi:10.1523/ENEURO.0026-25.2025)

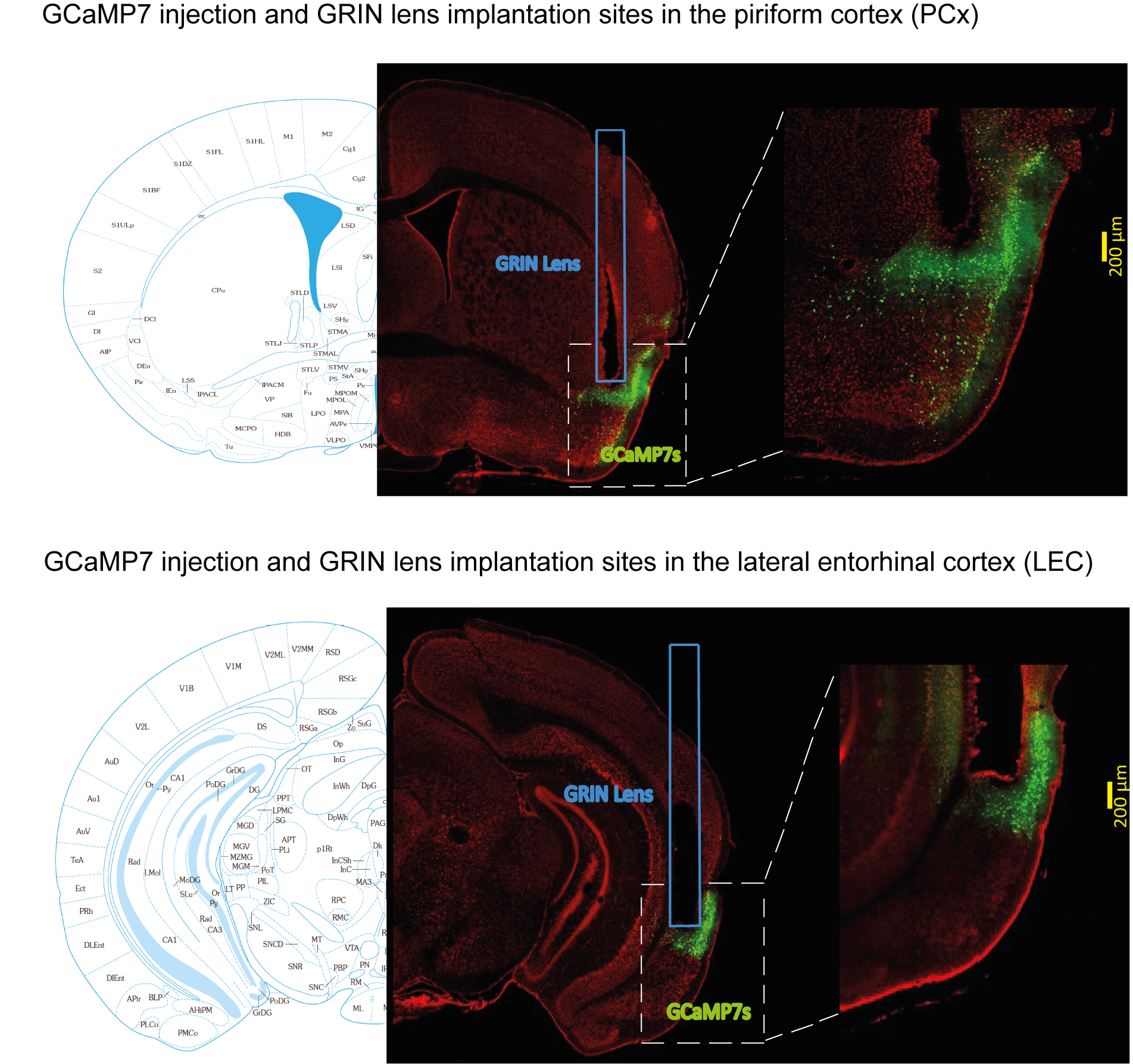

Supplement: Figure 1-1 — GCaMP7 injection and GRIN lens implantation sites in LEC and PCx Top left: Atlas schematics of coordinates of viral injection and GRIN lens implantation for the piriform cortex. Top middle: Representative GCaMP7s injection and GRIN lens implantation sites in the piriform cortex. Top right: An enlarged view of the GCaMP7s injection and GRIN lens sites in the piriform cortex. Scale bar: 200 µm represents the approximate imaging distance below the GRIN lens. Bottom left: Atlas schematics of coordinates of viral injection and GRIN lens implantation of the LEC. Bottom middle: Representative GCaMP7s injection and GRIN lens implantation sites in the LEC. Bottom right: An enlarged view of the GCaMP7s injection and GRIN lens sites in LEC. Scale bar: 200 µm represents the approximate imaging distance below the GRIN lens. Download Figure 1-1, TIF file. [file eneuro-12-ENEURO.0026-25.2025-s001.tif]

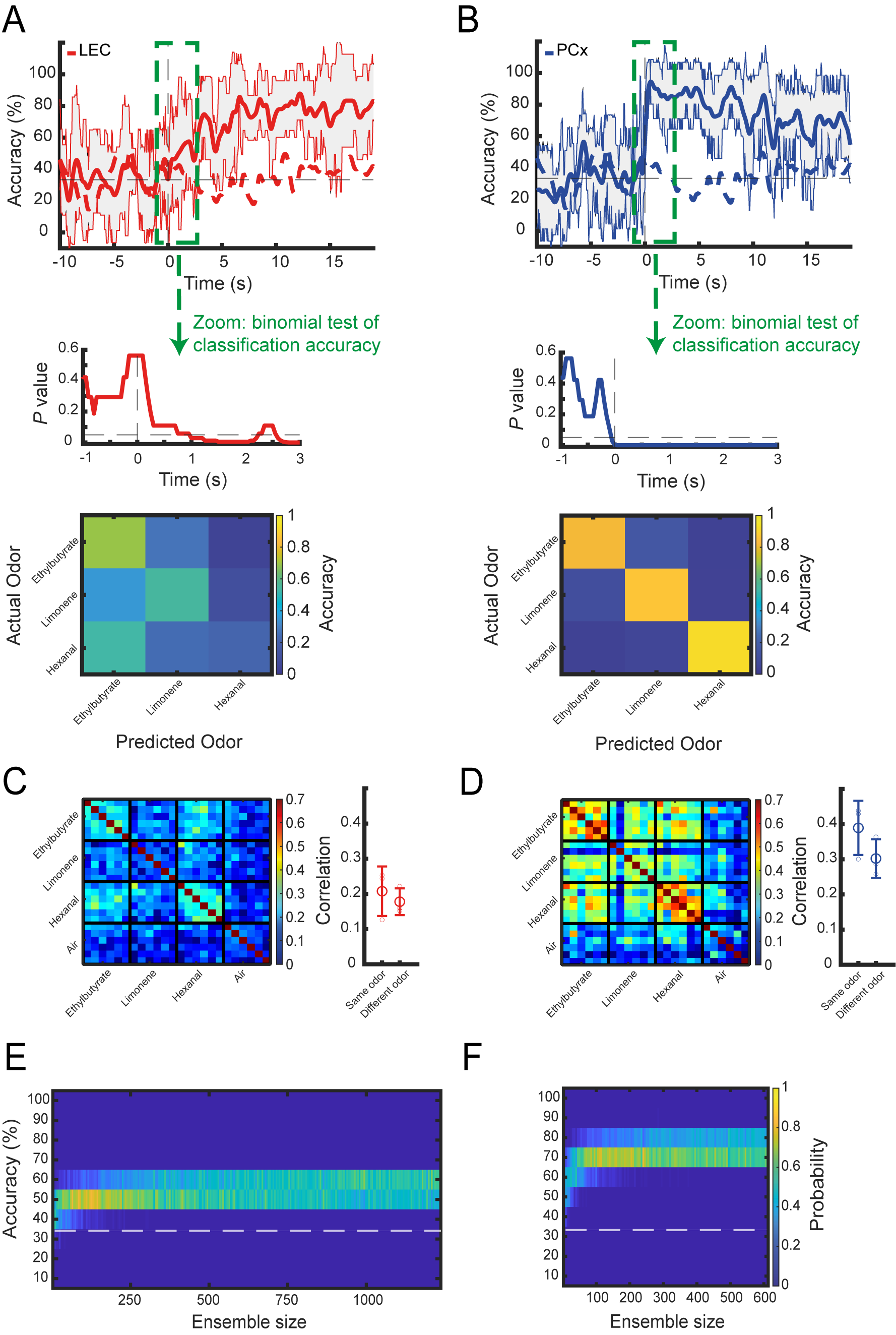

Supplement: Figure 2-1 — Dynamics of odor identity encoding in LEC and PCx in head fixed (A, B) Top panel: Accuracy of odor identity classification in LEC (A) and PCx (B) over time, for head-fixed mice. Time 0 indicates odor valve opening (vertical gray dashed line). Odor encoding is less accurate and delayed in LEC compared to PCx. The horizontal gray dashed line represents the chance level (1/3). Shaded area indicates 95% confidence intervals for the mean. Middle panel: Time-resolved binomial test of classification accuracy. The plots show the evolution of p-values over time (computed in time frames of 250 ms), testing whether decoding performance exceeds the chance level of 1/3. The vertical gray dashed line indicates odor valve opening (time = 0), and the horizontal gray dashed line marks the significance threshold (p = 0.05). Curve segments falling below this threshold indicate periods of statistically significant decoding. In (A), the LEC curve shows a ∼1-second delay before decoding becomes significant, whereas in (B), the PCx curve shows that odor encoding becomes significant immediately after odor presentation, with no apparent delay. Bottom panel: Confusion matrix summarizing the performance of the SVM classifier trained to discriminate the odorants. Decoding accuracy for each odor, averaged over a 2-second time window. LEC’s confusion matrix shows that classifier accuracy for ethylbutyrate reaches 69%, while limonene is classified at 54%. This accuracy for PCx is above 81% for all 3 odors. (C) Left: Similarity matrix representing the pairwise correlation coefficients between neuronal activity population response vectors in LEC. Data obtained from 6 pooled mice (5 sec odor-exposure window). Every small square represents a trial. A group of 6 trials constitutes an odor. Right: correlation of odor responses between repeat exposure to the same versus different odorants. Large circles: average, small circles: data points from individual mice; bar: standard deviation. (D) Left: Similar [file eneuro-12-ENEURO.0026-25.2025-s002.tif]

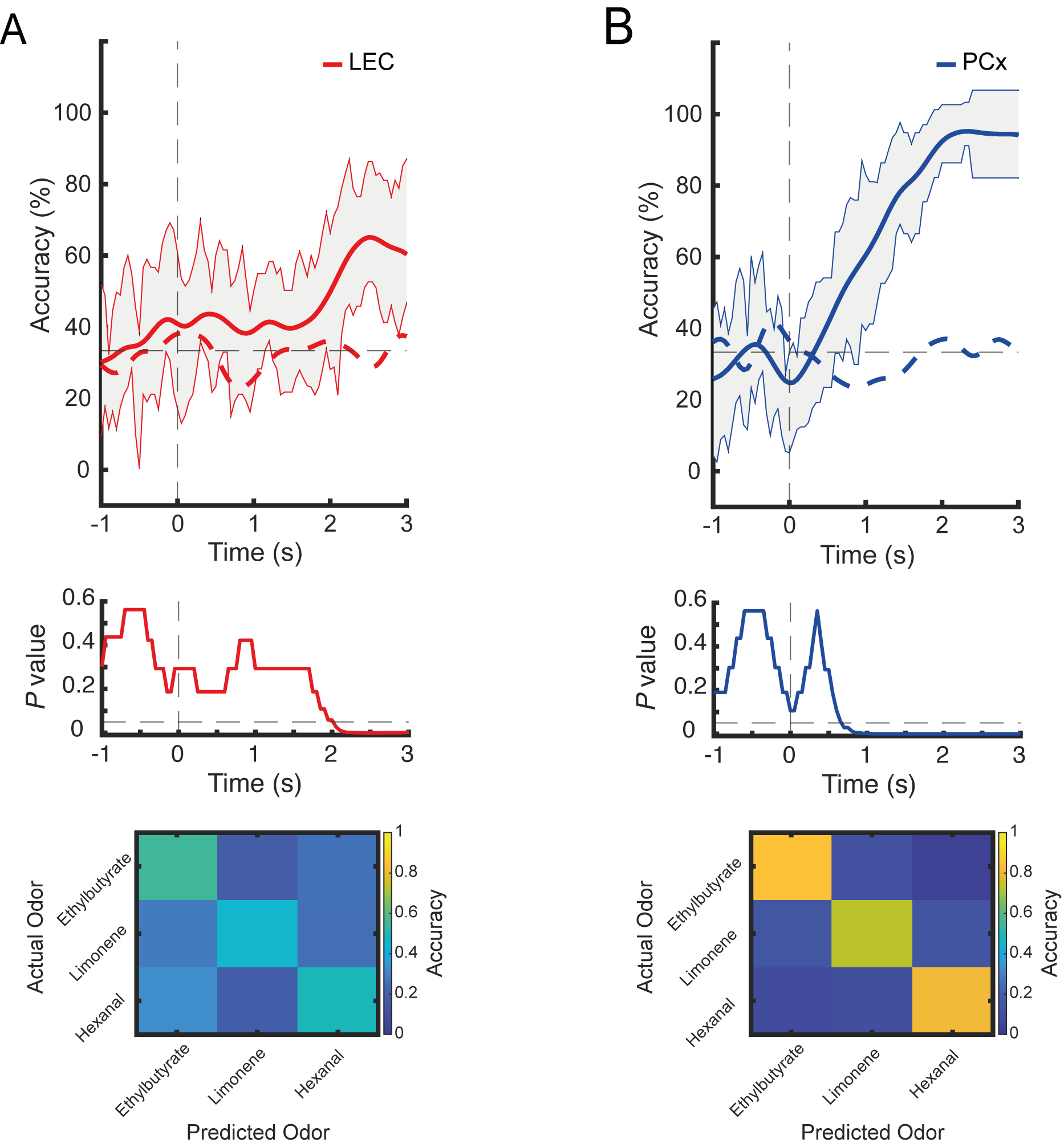

Supplement: Figure 3-1 — Dynamics of odor identity encoding in LEC and PCx in freely moving (A, B) Top panel: Accuracy of odor identity classification in LEC (A) and PCx (B) over time, for freely-moving mice. Time 0 indicates odor valve opening (vertical gray dashed line). Odor encoding is less accurate and delayed in LEC compared to PCx. The horizontal gray dashed line represents the chance level (1/3). Shaded area indicates 95% confidence intervals for the mean. Middle panel: Time-resolved binomial test of classification accuracy. The plot shows the evolution of the p-value over time (computed in time frames of 250 ms), testing whether decoding performance exceeds the chance level of 1/3. The vertical gray dashed line indicates odor valve opening (time = 0), and the horizontal gray dashed line marks the significance threshold (p = 0.05). Curve segments falling below this threshold indicate periods of statistically significant decoding. In (A), the LEC curve shows a ∼2-second delay before decoding becomes significant, whereas in (B), the PCx curve shows a shorter delay of 0.65 seconds before odor encoding becomes significant. Bottom panel: Confusion matrix summarizing the performance of the SVM classifier trained to discriminate the odorants. Decoding accuracy for each odor, averaged over a 2 second time window. The confusion matrix shows that classifier accuracy for ethylbutyrate reaches 55%, while limonene is classified at 45%, further supporting that odor-specific signals are present in LEC. This accuracy for PCx is above 90% in case of ethylbutyrate and hexanal, and 70% for limonene. Download Figure 3-1, TIF file. [file eneuro-12-ENEURO.0026-25.2025-s003.tif]
